# Improved Survival With Chemotherapy in Patients With Malignant Biliary Tract Obstruction After Percutaneous Transhepatic Biliary Drainage (PTBD)

**DOI:** 10.7759/cureus.63218

**Published:** 2024-06-26

**Authors:** Vikas K Jagtap, Sumit Kumar, Caleb Harris, Donboklang Lynser, Vandana Raphael

**Affiliations:** 1 Radiation Oncology, North Eastern Indira Gandhi Regional Institute of Health and Medical Sciences, Shillong, IND; 2 Surgical Oncology, North Eastern Indira Gandhi Regional Institute of Health and Medical Sciences, Shillong, IND; 3 Radiodiagnosis, North Eastern Indira Gandhi Regional Institute of Health and Medical Sciences, Shillong, IND; 4 Pathology, North Eastern Indira Gandhi Regional Institute of Health and Medical Sciences, Shillong, IND

**Keywords:** percutaneous transhepatic biliary drainage (ptbd), obstructive jaundice, hepato-pancreatico-biliary cancers, chemotherapy, malignant biliary tract obstruction

## Abstract

Introduction

Biliary tree stenting for malignant biliary tract obstructions is a routine modality for the relief of jaundice. Treatment is palliative in most circumstances. However, adequate reduction in bilirubin levels after percutaneous transhepatic biliary drainage (PTBD) may help to offer chemotherapy, which may improve survival in a limited number of cases.

Materials and methods

Between March 2017 and March 2023, patients who were treated with PTBD to relieve malignant biliary tract obstruction were included in the analysis. Patients who achieved bilirubin levels ≤5 mg/dL after PTBD were considered for chemotherapy. For survival analysis, a comparison was done between patients treated with chemotherapy after PTBD versus patients who did not receive any treatment after PTBD.

Results

Data was available for 43 (100%) patients. After PTBD, 16 (37.2%) patients responded and were considered for further treatment. One patient who was advised of radical surgery refused treatment and did not return for further treatment or follow-up. The remaining 15 cases (34.9%) received Gemcitabine and platinum-based chemotherapy as a first-line option. Out of 15 cases who received chemotherapy only one patient (6.6%) received neoadjuvant chemotherapy and the rest of 14 (32.5.%) cases received palliative chemotherapy in view of metastatic disease. PTBD complications including leakage, dislodgement of PTBD catheter, pain, and bleeding were seen in 16 (37.2%) cases. Overall survival was 57% for the entire population. Patients treated with chemotherapy after PTBD had better overall survival compared to patients who did not receive any treatment after PTBD (73.3% vs 33% (p=0.008)).

Conclusion

PTBD is an excellent technique for the relief of biliary obstruction. More than one-third (34.9%) of the cases received further cancer-directed treatment after relief of jaundice by PTBD. Chemotherapy after PTBD is associated with improvement in overall survival in malignant biliary obstructions.

## Introduction

Hepato-pancreatico-biliary tree tumors often present with malignant biliary tract obstruction as a primary presentation. Cancers of the gall bladder, pancreas, cholangiocarcinoma, and even external compression by tumor or nodal mass can cause biliary tract obstruction. Biliary tree stenting with the help of percutaneous transhepatic biliary drainage (PTBD) or endoscopic retrograde cholangiopancreatography (ERCP) are routine treatment modalities for the relief of obstructive jaundice [1]. PTBD is an effective technique in relieving biliary obstruction. Symptomatic relief from jaundice is the main benefit after PTBD or ERCP. Survival improvement is documented after relief of jaundice even without chemotherapy [2]. However, minimal information is available on the possibility of further oncological treatment and survival in this group of patients after PTBD. The hepatic metabolism of some chemotherapeutic drugs may preclude the possibility of such treatment in some cases [3]. High bilirubin levels along with poor performance status may deter the oncologists from offering any treatment. Treatment is palliative in most circumstances. However, adequate reduction in bilirubin levels after PTBD may help in treating patients with chemotherapy, which may improve survival in a limited number of cases. Stage, general condition, and various other factors may adversely affect the prognosis irrespective of PTBD [2]. Post-procedure complications including PTBD leakage, pain at the catheter site or in the right hypochondriac region, bleeding, and infection may affect the overall quality of life and treatment considerations [1]. This retrospective analysis was done to assess the benefit of chemotherapy in patients with malignant biliary tract obstruction who were treated with PTBD. Our hypothesis was that if PTBD can allow chemotherapy administration in patients with hyperbilirubinemia after a significant reduction in Bilirubin levels, these patients will have better survival compared to patients who were not fit for chemotherapy after PTBD. We analyzed the benefit of chemotherapy in comparison to no treatment in patients with malignant biliary tract obstruction following PTBD.

## Materials and methods

Between March 2017 and March 2023, patients who were treated with PTBD to relieve malignant biliary tract obstruction at our institute were included in the analysis. In this retrospective study, patients who were diagnosed to have malignant biliary obstruction due to any malignancy, including hepato-pancreatico-biliary cancer, cancers of other origins, or metastatic tumor masses, were also included in the analysis. For case selection, Multidisciplinary Tumor Board (MDT) data was reviewed to select cases who were advised PTBD for relief of biliary tract obstruction. Patient file records were obtained from the medical records section for analysis of the data for patients who underwent PTBD procedure as per MDT decisions. Only patients for whom medical records were available were included in the analysis. Since ERCP is not available at our institute, only cases who consented to external drainage with PTBD after MDT decisions and who were further treated with chemotherapy at our institute were included in the analysis. In our institute, all PTBD procedures are performed by a trained intervention radiologist team with the patient under local anesthesia or sedation. A Pigtail or Malecot catheter of size 8-10 Fr (French gauge size) is routinely used at our institute for PTBD drainage procedures. All PTBD procedures are carried out under ultrasonography or fluoroscopy guidance. Demographic and patient-specific data, like age, sex, pre-procedure Eastern Cooperative Oncology Group Performance Score (ECOG-PS), cancer type, histology, various comorbidities, and total serum bilirubin levels in mg/dL, were recorded for study purposes [4]. As per our institute protocol, total bilirubin level ≤5 mg/dL after PTBD was considered as a criterion for the administration of chemotherapy in patients with ECOG-PS: 0-1. The highest bilirubin levels prior to the procedure and the time (number of days) taken to achieve a total bilirubin level ≤5 mg/dL were recorded. Blood reports; histopathology reports; and PTBD complications like pain, bleeding, and catheter site leak were documented and used for analysis. Respondents were defined as those with a reduction in bilirubin level ≤5 mg/dL after the PTBD procedure. For survival analysis, patients were stratified between two groups: patients who received chemotherapy after PTBD and patients who were advised best supportive care or received no treatment after PTBD. Survival time was calculated from the PTBD procedure date up to the last follow-up date or patient’s death.

For the literature review, relevant data from previously published studies and abstracts were reviewed. A Medline/Pubmed search was done for the literature review. The study was reviewed by the institutional scientific advisory committee and was approved by the Institutional Ethics Committee (IEC). The patient consent was waived by IEC in view of the retrospective nature of the analysis. Microsoft Excel and IBM SPSS Statistics V 29.0.2.0 (20) software were used for data entry and analysis. For survival analysis, the Kaplan-Meier method was used, and the Chi-square test (log-rank) was used for p-value determination.

## Results

**Figure 1:**
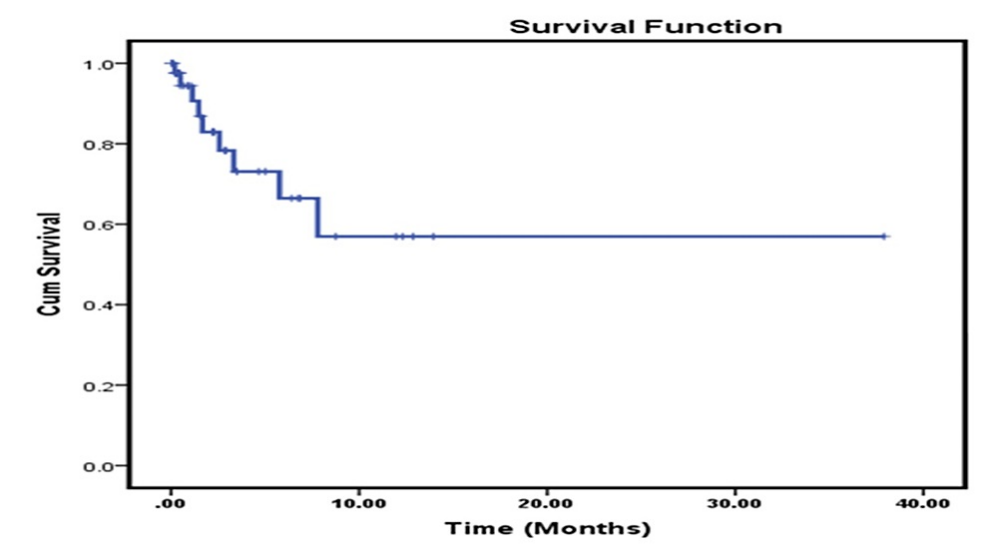
Overall survival

**Figure 2:**
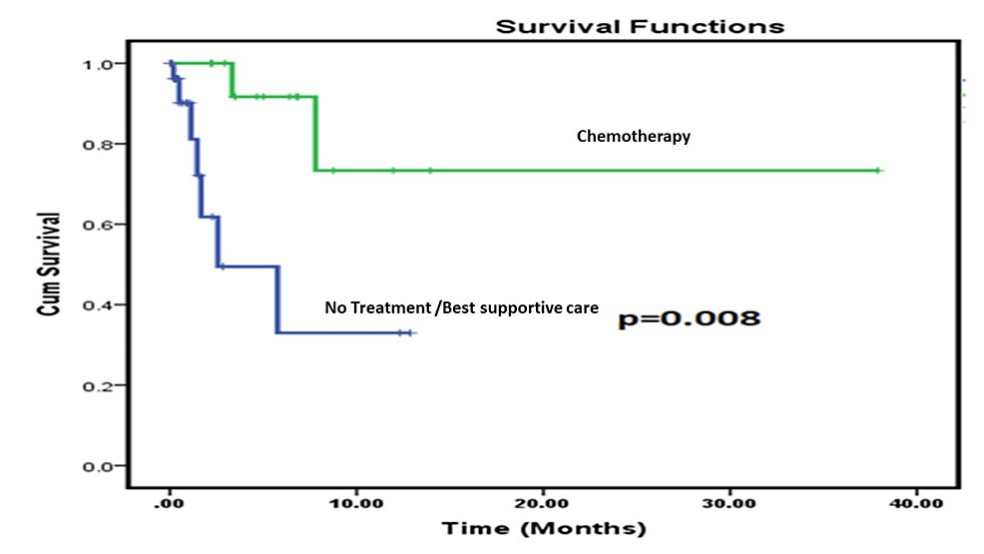
Overall survival in patients who received treatment (chemotherapy) after PTBD versus patients who received no treatment after PTBD

Data was available for 43 (100%) patients. The demographic profile and diagnostic details are shown in Table 1. Pain in the abdomen, yellowish discoloration of skin/urine, and itching over the body were the most common presentations. Table 2 shows the treatment-related parameters. After PTBD, 16 (37.20%) patients responded and were considered for further treatment. Out of 16 cases who responded, one patient (2.3%) who was advised for surgery did not return for further treatment or follow-up. The remaining 15 cases (34.9%) received Gemcitabine/platinum-based chemotherapy as a first-line option. Out of 15 cases who received chemotherapy only one patient (2.3%) received neoadjuvant chemotherapy and the remaining 14 (32.5 %) received palliative chemotherapy in view of metastatic disease. CAPEOX (Capecitabine and Oxaliplatin) or FOLFOX (Fluorouracil (5-FU), Leucovorin calcium and Oxaliplatin) was given as the second-line option for four cases who had progressive disease after first-line chemotherapy. The remaining 28 (65.1%) patients did not receive any further treatment due to various reasons including patients with poor ECOG PS, refusal of treatment, and total bilirubin level ≥5 mg/dL after PTBD. In 11 (25.5%) cases, bilirubin levels were still high (>5 mg/dL) after the PTBD procedure out of which five patients expired, two (4.7%) cases expired after PTBD procedure before any treatment can be considered (one with ECOG PS: 3), one case (2.3%) due to poor ECOG PS, three (6.9%) cases were advised for best supportive care as per clinician’s discretion in view of poor GC after PTBD, three (6.9%) cases refused treatment, and remaining patients were lost to follow up after PTBD. Out of 43 (100%) patients, a total of nine (20.93%) patients expired, five (11.6%) in the no treatment group, and two (4.7%) in the treatment group, all within six months of PTBD. The remaining 30 (69.8%) patients were lost to follow-up. Only one case (2.3%) is still on active treatment with palliative chemotherapy at the time of analysis. Mean overall survival was 57% (Figure 1) for the entire population. On subset analysis, patients who received chemotherapy (N=15 (34.9%)) after PTBD had better overall survival compared to patients who received no treatment (N=28 (65.1%)) or only best supportive care, 73.3% vs 33% (p=0.008, log-rank test, Figure 2). Mean overall survival was 23.2 months (15.09-31.27)±4.12 for all patients. Patients who received treatment had a mean overall survival of 29.5 months (18.84-40.15)±5.43 compared to those who did not receive any treatment 6 months (2.61-9.29)±1.70.

**Table 1:** Patient demographic data PTBD, percutaneous transhepatic biliary drainage; FNAC, fine needle aspiration cytology; ECOG PS, Eastern Cooperative Oncology Group Performance Status

Demographic parameters		N-43 (100%)
Age, median (range)	54 (19-84)	-
Sex	Male	19 (44.1%)
	Female	24 (55.9%)
Comorbidities	No comorbidities	25 (58.20%)
	Diabetes, hypertension	4 (9.30%)
	Not known	14 (32.5%)
ECOG PS	1	31 (72%)
	2	10 (23.3%)
	3	2 (4.7%)
Method of diagnosis	FNAC	33 (76.8%)
	Biopsy	8 (18.6%)
	Fluid cytology - from PTBD	2 (4.6%)
Diagnosis	Gall bladder cancer	28 (65.1%)
	Cholangiocarcinoma	11 (25.6%)
	Pancreatic cancer	1 (2.3)
	Hepatocellular carcinoma	2 (4.7%)
	Gastroesophageal junction cancer	1 (2.3%)
Nonmetastatic	Gall bladder cancer	3 (6.9%)
	Cholangiocarcinoma	1 (2.3%) - expired without treatment
Metastatic	Liver metastasis	19 (48.8%)
	Lung metastasis	4 (10.3%)
	Nonregional lymph nodes	11 (28.2%)
Histopathology	Adenocarcinoma	39 (90.7%)
	Hepatocellular carcinoma	2 (4.7%)
	Squamous cell carcinoma (from Gastroesophageal Junction)	1 (2.3%)

**Table 2:** Treatment-related parameters

Treatment parameter	N (%)
Chemotherapy	15 (34.9%)
Best supportive care or no treatment	28 (65.1%)
PTBD complications - (leakage/dislodgement/pain/bleeding), N-16 (37.2%)	10 (23.25%) - chemotherapy group
	6 (13.95%) - no treatment/best supportive care group
Bilirubin level before PTBD - mean (range)	19.24 (7.9-45.8)
Bilirubin level after PTBD at last follow up - mean (range)	9.25 (0.6-28.8)
Time for bilirubin level to reach below 5 mg/dL (days) - median (range)	30 (7-90)

## Discussion

Malignant biliary tract obstructions are commonly seen in hepato-pancreatico-biliary tract tumors. Evidence supports that patients with ECOG: 0-1, chemotherapy administration, bilirubin level <120 µmol/L, and serum LDH levels <300 µmol/L were associated with improved survival after PTBD [5]. Adequate decline in bilirubin levels may help the patients symptomatically and may also make them fit for further chemotherapy [6]. Cancers other than hepato-pancreatico-biliary tree have also been reported to benefit from PTBD [7,8]. In our study, more than 95% of patients had cancer of the hepatic-pancreatico-biliary tree and only one case had biliary obstruction due to metastatic nodal mass arising from gastroesophageal junction cancer. Pain at the catheter site or in the abdomen, bile leakage, frank bleeding, infection of the biliary or pancreatic system, and stent dysfunction are the various risks or complications associated with biliary stenting with PTBD [1]. In our experience, 16 (37.20%) of our patients had various PTBD-related complications including leakage, catheter dislodgement, pain at the catheter site and right hypochondriac region, and bleeding. All these complications were managed conservatively with reinsertion/repositioning of PTBD or internalization of PTBD catheter.

The main strength of our study is the consideration of chemotherapy for the patients who responded well to PTBD and were considered for chemotherapy even without normal bilirubin levels. At our institute, total bilirubin level ≤5 mg/dL after PTBD was considered as a criterion for chemotherapy consideration in patients with good general condition, i.e., PS: 0-1. The mean total bilirubin was 14.9 (range: 8.3-20.4) prior to PTBD for patients who received chemotherapy. As per our analysis in more than one-third or 34.9% of our patients, we were able to achieve beneficial ≤5 mg/dL levels of bilirubin after PTBD to be considered for chemotherapy as per our institute criteria.

Survival advantage in patients who underwent PTBD has been documented by Niemelä J et al. [9]. The authors found 11.7 months of median survival in 32 (20.3%) patients who underwent chemotherapy compared to a median survival of 1.7 months in 126 (79.7%) patients who received only the best supportive care. The overall survival was significantly better in patients who underwent chemotherapy after PTBD (P<0.001) [9]. As per our study, overall survival benefit (p=0.008) was seen in patients who underwent chemotherapy after PTBD. In our study, overall survival was 29.5 months vs six months in patients who underwent chemotherapy compared to patients who had no treatment after PTBD.

We also think that fit patients (PS: 0-2), with higher bilirubin levels (5-10 mg/dL) with mild hepatic dysfunction may also be given a benefit of chemotherapy. In our study, most of the patients (40 (93%)) received Gemcitabine and platinum-based regimens as first-line chemotherapy in view of a higher percentage of gall bladder cancers (65.1%), cholangiocarcinoma (25.6%), and pancreatic cancer (2.3%). Pelzer U et al. demonstrated that overall survival did not differ between the three groups who had bilirubin levels of 1.2-3 mg/dL, >3-5 mg/dL, and >5 mg/dL after the first application of Nabpaclitaxel and Gemcitabine-based chemotherapy [10]. Joerger M et al also proposed that in advanced pancreatico-biliary cancers, Gemcitabine and Capecitabine can be safely administered with hepatic dysfunction and high bilirubin level [11]. However, this hypothesis needs to be tested in a prospective setting for a definitive answer. Many oncologists differ chemotherapy in patients with PS: 0-2 and high bilirubin levels. Julia Quidde et al. in their series of 12 patients with metastatic liver disease demonstrated that chemotherapy with Fluorouracil (5-FU) and Oxaliplatin is feasible even with deranged liver functions and hyperbilirubinemia [12]. The authors also demonstrated the safety of addition of the monoclonal antibodies like trastuzumab, bevacizumab, and cetuximab along with chemotherapy in such cases. Batra et al. demonstrated the feasibility of a FOLFOX-based regimen in patients with hyperbilirubinemia in advanced pancreato-biliary cancers [13]. Jun Gong et al. proposed that a dosing modification strategy with chemotherapeutic drugs is a rational approach in patients with liver dysfunction and hyperbilirubinemia [14]. Our results support the fact that chemotherapy drugs like Gemcitabine, platinum, 5FU, Capecitabine, and Oxaliplatin can safely be given in such cases for better outcomes. Liver dysfunction and hyperbilirubinemia should not deter oncologists from considering chemotherapy in selected cases taking into consideration the performance score and comorbidities. We also advise that in patients with hyperbilirubinemia, an option for biliary drainage with stenting or PTBD should be considered before chemotherapy.

Although the majority of our cases were metastatic, four nonmetastatic patients were also included. One patient had a poor general condition (ECOG PS: 3) so only the best supportive care was advised. One patient refused treatment. One case was planned for assessment after neoadjuvant chemotherapy but developed liver metastasis and one case expired without any treatment. 

Furthermore, some of our cases not fit for chemotherapy may also be given the benefit of intraluminal brachytherapy (ILBT). Sood et al. demonstrated overall survival benefit at six months with 62.34% overall survival in the PTBD plus ILBT group compared to 3.64% (p<0.0001) in the PTBD alone group [15]. We could not assess the benefit of ILBT in our cases as brachytherapy services were not available at our center during the study period. The study is retrospective and includes patients with heterogeneity of disease location, histology, and stage. This is an inherent flaw but it does not hamper the key reasoning that PTBD helps in decreasing hyperbilirubinemia to a level where chemotherapy can be delivered.

## Conclusions

PTBD is an excellent technique to reduce bilirubin levels in malignant biliary tract obstructions. Chemotherapy benefits should be offered to patients after PTBD with a fall in bilirubin levels and with good performance status. PTBD is effective in facilitating the delivery of chemotherapy in 34.9% of patients in the real-world scenario. Chemotherapy can be safely administered with hyperbilirubinemia after PTBD with a bilirubin level ≤5 mg/dL. Further research in patients with bilirubin levels >5 mg/dL will help to benefit these cases, which are otherwise considered palliative.
